# Serum miR371 in testicular germ cell cancer before and after orchiectomy, assessed by digital-droplet PCR in a prospective study

**DOI:** 10.1038/s41598-021-94812-2

**Published:** 2021-08-02

**Authors:** Mette Pernille Myklebust, Anna Thor, Benedikte Rosenlund, Peder Gjengstø, Ása Karlsdottir, Marianne Brydøy, Bogdan S. Bercea, Christian Olsen, Ida Johnson, Mathilde I. Berg, Carl W. Langberg, Kristine E. Andreassen, Anders Kjellman, Hege S. Haugnes, Olav Dahl

**Affiliations:** 1 Mohn Cancer Research Laboratory, Department of Oncology and Medical Physics, Haukeland University Hospital, Jonas Lies vei 91B, 5021 Bergen, Norway; 2 Department of Urology and CLINTEC Karolinska Institutet, Karolinska University Hospital, Stockholm, Sweden; 3 Department of Urology, Haukeland University Hospital, Bergen, Norway; 4 Department of Urology, Helse Fonna, Haugesund, Norway; 5 Department of Urology, Oslo University Hospital, Oslo, Norway; 6 The Cancer Centre, Oslo University Hospital, Oslo, Norway; 7 Department of Oncology, University Hospital of North Norway, Tromsø, Norway; 8 Department of Clinical Medicine, UIT-The Arctic University, Tromsø, Norway

**Keywords:** Cancer, Biomarkers, Urology

## Abstract

MicroRNA-371a-3p (miR371) has been suggested as a sensitive biomarker in testicular germ cell cancer (TGCC). We aimed to compare miR371 with the classical biomarkers α-fetoprotein (AFP) and β-human chorionic gonadotropin (hCG_β_). Overall, 180 patients were prospectively enrolled in the study, with serum samples collected before and after orchiectomy. We compared the use of digital droplet PCR (RT-ddPCR) with the quantitative PCR used by others for detection of miR371. The novel RT-ddPCR protocol showed high performance in detection of miR371 in serum samples. In the study cohort, miR371 was measured using RT-ddPCR. MiR371 detected CS1 of the seminoma and the non-seminoma sub-types with a sensitivity of 87% and 89%, respectively. The total sensitivity was 89%. After orchiectomy, miR371 levels declined in 154 of 159 TGCC cases. The ratio of miR371 pre- and post-orchiectomy was 20.5 in CS1 compared to 6.5 in systemic disease. AFP and hCG_β_ had sensitivities of 52% and 51% in the non-seminomas. MiR371 is a sensitive marker that performs better than the classical markers in all sub-types and clinical stages. Especially for the seminomas CS1, the high sensitivity of miR371 in detecting TGCC cells may have clinical implications.

## Introduction

Testicular germ cell cancer (TGCC) is the most common cancer type among young men aged 15–49 years in the Western countries. Briefly, post-pubertal TGCC derived from germ cell neoplasia in situ (GCNIS) is classified into the histological sub-types seminomas and non-seminomas, with non-seminomas further classified into embryonal carcinomas, choriocarcinomas, yolk-sac tumours and teratomas^1^. The disease is effectively treated surgically with the addition of chemotherapy in high-risk clinical stage 1 (CS1) and metastatic cases^2^. Chemotherapy is administered according to TGCC sub-type and stage at diagnosis and is in Norway and Sweden risk-adapted in order to reduce the amount of chemotherapy given to these young patients^3^. Unfortunately, this treatment success comes with a price as the cytotoxic treatment is associated with severe long-term and late side effects e.g. impaired infertility, peripheral neuropathy, second cancers^4^ and cardiovascular disease^5–8^, and also increased late mortality manifested up to 30 years after treatment^9–11^. The toxicity of the cisplatin-based cycles used is additive^12,13^; hence there is a large benefit by avoiding overtreatment in this patient group.

The current circulating biomarkers used in management of TGCC are human chorionic gonadotropin type beta (hCG_β_), alpha-fetoprotein (AFP) and lactate dehydrogenase (LDH). One challenge is that these biomarkers, particularly LDH, have low sensitivity and specificity. Overall, AFP and hCG_β_ are expressed at pathological levels in less than 50% of all testicular TGCC cases^14^. Especially for the teratomas and pure seminomas there is an evident need for new, circulating markers.

MicroRNAs (miRs) are small, endogenous and non-coding RNAs that are involved in posttranscriptional regulation of gene expression^15^. MicroRNAs of the miR-302/367 and miR371-373 clusters were found to be expressed in embryonal stem cells^16^ and in testicular germ cell tumours^17^. Later, circulating microRNA-371a-3p (miR371) has been identified as the most promising candidate and shown to be present in almost all TGCC patients^17–23^. A large, prospective multicentre study showed miR371 to be positive in 90.1% of the TGCC cases at the time of diagnosis^20^. The specificity of the test was also very high (94%). Recently, this superior sensitivity and specificity was reproduced by another group^24^. Studies also showed that miR371 levels were elevated in cases with residual tumour masses after chemotherapy and in recurrent cases^20,24,25^. Detection of circulating miR371 in serum or plasma are challenging as the levels are very low. The published studies have all used detection of miR371 by quantitative reverse transcriptase PCR (RT-qPCR) and hydrolysis probes after microRNA specific cDNA synthesis. A pre-amplification has been added to the protocol as the levels are approaching the limits for detection by RT-qPCR^26,27^. Digital droplet PCR (ddPCR) has been increasingly used the last years, also for microRNA analysis^28,29^. ddPCR has been shown to have greater precision and less day-to-day variation compared to RT-qPCR, especially at very low abundance of target molecules^28,30,31^.

MicroRNAs were initially presumed to be quite stable at room temperature in serum, an important feature for a clinical biomarker^27^. However, studies indicate microRNAs in blood to be altered during storage^32–35^. Hence, the stability of each microRNA must be established specifically.

In the present study, TGCC patients undergoing orchiectomy were included prospectively and serum miR371 was analysed in pre- and post-orchiectomy samples. We aimed to verify the high sensitivity of miR371 by reverse transcription digital droplet PCR (RT-ddPCR) in detection of TGCC as reported by others using RT-qPCR. We compared the performance of reverse RT-ddPCR with RT-qPCR and assessed the sensitivity and specificity of miR371 as a biomarker for TGCC. We also present data indicating the stability of serum miR371 in vitro and the half-life of miR371 in vivo.

## Results

### Performance of the qPCR and ddPCR miR371 protocols

For RT-qPCR, the no-template control (NTC) consistently showed no amplification signal (Cq > 40), and the Negative serum control had Cq values in the range 30.5 to > 40. Correspondingly, for RT-ddPCR the number of positive droplets in NTC and the negative serum control was well below the technical threshold of at least 3 positive droplets in 2 out of 3 replicates in all runs. One or two positive droplets were detected in the NTC in eight out of 60 runs. For the negative serum control, the mean value was 0.92 droplets (95% CI 0.69–1.15, range 0–4 droplets for the triplicate wells).

**Figure 1:**
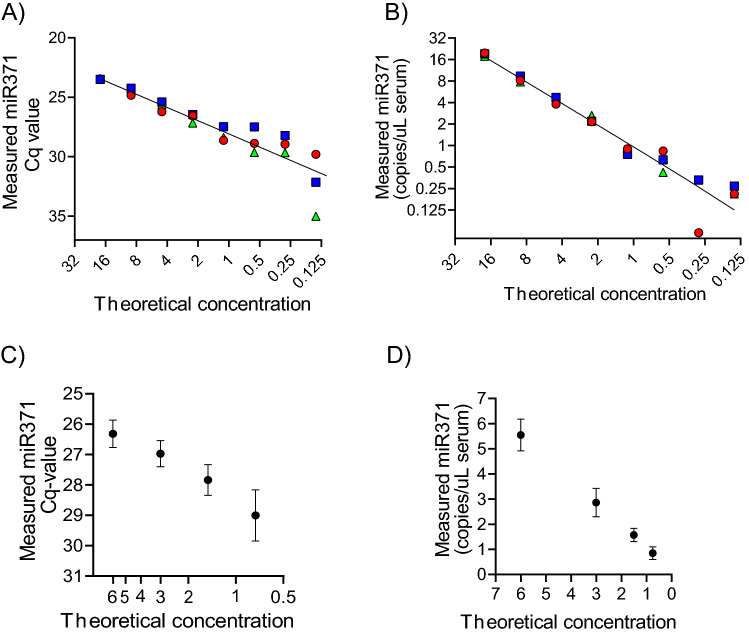
Quantification of miR371 by RT-qPCR and RT-ddPCR in dilution series. Twofold dilutions of miR371 quantified by (**A**) RT-qPCR and (**B**) RT-ddPCR. Each colour and shape represent an independent dilution curve preparation. (**C**) Repeatability at the lower concentration range for (**B**) RT-qPCR and (**D**) RT-ddPCR. The results for each dilution are shown as mean of ten measurements with standard deviation. Note that the results are given as raw Cq-values for RT-qPCR, but as copies/µL serum for RT-ddPCR. Due to the logarithmic nature of the RT-qPCR results, a difference of 1.0 Cq-value represents a twofold change in miR371. Also note that the direction of the axes is reversed for RT-qPCR in order to facilitate comparison to ddPCR.

Our results show the RT-qPCR miR371 assay to be linear from our highest included concentration of Cq-value 23.45 to Cq-value 28.15 (Fig. 1A). RT-ddPCR showed a comparable linearity from the highest included value of 18.90–0.82 copies/µL serum (Fig. 1B). The amplification efficiency for miR371 measured by qPCR with preamplification was 87% compared to 93% for ddPCR. The variation at the lower concentrations is shown for qPCR and ddPCR in Fig. 1C,D, respectively. The LOD determination for qPCR by repeated measurement of a sample with Cq-value first identified to be 30.5, showed that results of samples with Cq > 30 was not reproduced (Supplementary Information and Data 3). Four of the blood donors had positive amplification curves at Cq-values 31.8, 31.1, 30.9 and 30.3. These results taken together, the threshold for calling miR371 positive in patient samples using RT-qPCR with pre-amp was set to Cq ≤ 29.5.

The mean miR371-level of the blood donors analysed by RT-ddPCR was 0.03 copies/µL serum (95% CI 0.02–0.05), range 0.0–0.27 copies/µL serum. The miR371 ddPCR assay was linear down to measured 0.82 copies/µL serum. The LOB was calculated to be 0.13 copies/µL serum at the 99% confidence level from the blood donor samples. Calculated LOD was thus 0.29 copies/µL. Patients samples with miR371 values higher than 0.45 copies/µL serum were called as positive, given that the miR30b was > 300 copies/µL serum and the total number of droplets for each well was > 13,000. The threshold of 0.45 copies/µL serum was established from the calculated LOD and the technical limit of at least three positive droplets in two out of three replicates, which corresponds to a value of ~ 0.39 droplets/µL serum. The threshold is above the highest miR371 levels from the blood donors. The LOQ was identified to be > 0.85 copies/µL serum (Supplementary information and Data 4).

### Reproducibility and repeatability

The two runs in the reproducibility test produced comparable results, for both RT-ddPCR and RT-qPCR. Supplementary Figure 2(A) and (C) show that both techniques tend to measure higher levels in the second run for miR371. The bias can most probably be attributed to the cDNA synthesis^36^. The high concordance of miR30b between the two runs (Supplementary Figure 2B and D), indicates that both techniques have good reproducibility at higher abundance of the target. Further, when we examined the repeatability in three samples which expressed miR371 at varying levels, the CV for ddPCR were 34.5%, 20.6% and 14.4% from lowest to highest concentration respectively (Supplementary Figure 3A). The corresponding values for RT-qPCR were 2.7%, 1.24% and 0.08%. respectively (Supplementary Figure 3B). When assessing repeatability and reproducibility, CV (%) < 35% and < 3% was considered acceptable in quantitative analysis (copy number, RT-ddPCR) and in qualitative analysis (raw Cq, RT-qPCR), respectively.

We conclude that RT-qPCR with pre-amplification and RT-ddPCR both performs well with comparable results. RT-ddPCR was chosen as the preferred analysis method due to the its high performance without pre-amplification and the applicability of the analysis results as copies/µL serum in a clinical setting. The serum samples of the Study Cohort were analysed using RT-ddPCR.

### miR371 as a biomarker at primary diagnosis

**Figure 2:**
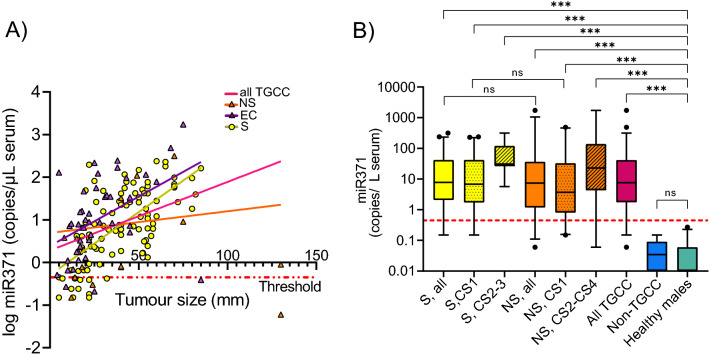
(**A**) Association between tumour size and miR371 expression in the histological subtypes of TGCC. The tumour size is plotted against miR371 expression. Regression lines for the subtypes are shown. A tumour was classified as EC if >20% of the tumour bulk consisted of EC cells. *NS* non-seminoma, *EC* embryonal carcinoma, *S* seminoma. (**B**) MiR371 expression prior to orchiectomy across the histological sub-types and clinical stages. The threshold for calling miR371 as positive is shown as a dotted, red line. Shown are mean values with 95% confidence interval. *S* seminomas, *NS* non-seminomas, *TGCC* testicular germ cell cancer, TGCCC, patients included with suspected TGCC, but diagnosed with other benign and malignant conditions; Healthy males, blood donors. ***P < 0.001; **P < 0.01; *P < 0.05; ^ns^*P* > 0.05.

Our study prospectively included 180 patients with samples pre- and post-orchiectomy. After orchiectomy, 21 were diagnosed with other diagnoses than TGCC, of these were 15 benign, three Leydig cell tumours, two B-cell lymphomas and one malignant leiomyosarcoma. Of the TGCCs, 97 were seminomas and 62 non-seminomas. The clinical and pathological variables for all patients are listed in Table 1. The median miR371 expression in the TGCC patients was 7.59 copies/µL serum, range 0.06–1734.0 copies/µL serum. Linear regression analyses showed an association between tumour size and miR371 serum levels for the whole TGCC cohort (R^2^ = 0.159, *P* < 0.001), the embryonal carcinomas (EC, R^2^ = 0.211, *P* < 0.001) and the seminomas (R^2^ = 0.433, *P* < 0.001), but not the non-seminomas including both mixed NS and EC (Fig. 2A).

**Table 1:** Clinicopathological variables for the participants of the study.

Parameter	Participants (N, %), total N = 180				
	CS1 (N = 131)^a^	CS2 (N = 25)^a^	CS3 (N = 1)^a^	CS4 (N = 2)^a^	Controls (N = 21)
Age (median, range)	35.3 (17.8–47.9)	32.5 (19.1–52.13)	32.8	36.1 (32.3–39.5)	39.0 (21.0–80.8)
**Histological type**					
Seminoma	86 (65.6)	10 (40.0)	1 (100)	0	–
Non-seminoma	45 (34.4)	15 (60.0)	0	2 (100)	–
Embryonal carcinoma	13 (9.9)	5 (20.0)	–	0	–
Yolk sac tumour	0	0	–	0	–
Choriocarcinoma	0	0	–	0	–
Teratoma^b^	1 (0.8)	1 (4.0)	–	0	–
Mixed non-seminoma	31 (23.7)	9 (36.0)	–	2 (100)	–
Benign/non-TGCC	–	–	–	–	21
Healthy males	–	–	–	–	50
Tumour size (cm) (median, range)	3.0 (0.4–8.5)	4.8 (0.5–13.0)	2.6 (NA)	3.6 (2.3–4.9)	–
Vascular invasion	20 (15.3)	14 (56.0)	1 (100)	2 (100)	–
AFP (above upper ref. limit)^c^	23 (17.7)	8 (32.0)	1 (100)	2 (100)	
HCG_β_ (above to upper ref. limit)^d^	46 (35.1)	14 (56.0)	1 (100)	2 (100)	
miR371 positive	115 (87.8)	24 (96.0)	1 (100)	2 (100)	0
miR371 copies/µL serum (mean, SD)	30.50 (62.69)	126.29 (344.46)	26.31 (–)	56.60 (73.67)	0.05 (0.05)

*N* number, *CS* clinical stage, *TGCC* testicular germ cell cancer, *AFP* alfa-fetoprotein, *hCG*_*β*_ human chorionic gonadotropin.

^a^Clinical stage is according to the Royal Marsden staging system^52^.

^b^Among the 180 included patients, two were pure teratomas, 34 had a teratoma component.

^c^Pre-orchiectomy AFP was missing for 5 patients.

^d^Pre-orchiectomy hCG_β_ was missing for 11 patients.

Both seminomas and non-seminomas CS 1–4 had statistically significant higher pre-orchiectomy miR371 levels than both healthy males and the included non-TGCC cases (Fig. 2B). There was no statistically significant difference in miR371 levels when seminomas were compared to non-seminomas. Overall, 17 (10.7%) TGCC patients were miR371 negative (six non-seminomas and eleven seminomas). None of these were pure teratomas. One of the negative non-seminoma patients was CS2 and had a 13 cm large tumour classified as 60% teratoma and 40% seminoma, while three were embryonal carcinoma CS1 and two were mixed non-seminomas CS1. All negative seminomas were classified as CS1. One of the miR371 negative patients had hCG_β_ slightly above the threshold, the other 16 were negative for both hCG_β_ and AFP.

The miR371 RT-ddPCR analysis with threshold 0.45 copies/µL serum had a sensitivity of 89% (95% CI 85–94%) and a PPV of 100% for detection of TGCC in the entire study cohort. The sensitivity for CS1–4 seminomas was 89% (95% CI 82–95%) and 0.90 (95% CI 83–98%) for CS1–4 non-seminomas. Seminomas CS1 and non-seminomas CS1 had sensitivities of 87% (95% CI 80–94%) and 89% (95% CI 80–98%), respectively. The specificity for the whole cohort was 100%. The NPV was 55% (95% CI 39–71%) for the entire cohort.

**Figure 3:**
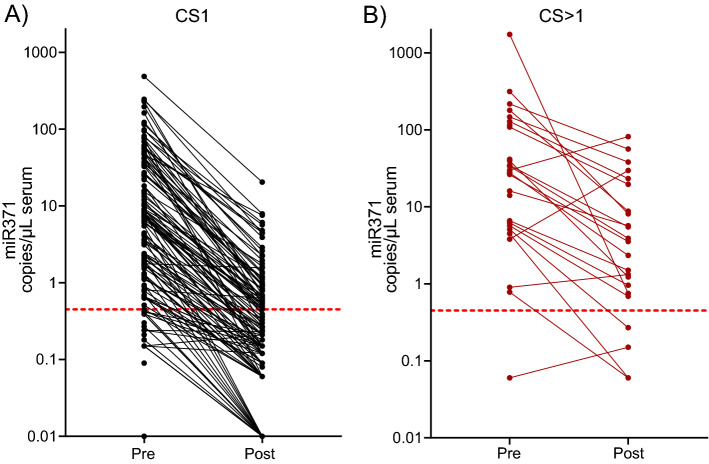
miR371 levels pre- and post-orchiectomy. The decrease in miR371 after orchiectomy in CS1 (N = 131, (**A**)) and for CS > 1 (N = 28, (**B**)). The red, dotted line marks the threshold for calling miR371 as positive.

Among the miR371 positive CS1 orchiectomised patients (N = 115), 97.9% had decreased miR371 levels 18–48 h after removal of primary tumour (Fig. 3A). For CS1 patients, the median ratio for miR371 values at baseline versus 18–48 h after orchiectomy was 20.5 (range 1.1–1405.7). For CS2–4 patients, 85.7% showed a decline in miR371 after orchiectomy, but the median ratio of 6.5 (range 0.13–74.87) was statistically lower than for CS1 (Fig. 3B, *P* = 0.011). MiR371 levels increased for three of the patients after orchiectomy with CS > 1, of whom two were CS 2 patients in which miR371 levels were normalised after start of chemotherapy, the third was a CS 2 patient where miR371 was normalised after RPLND and chemotherapy. Histological analysis showed EC in the RPLND specimen.

Two of the included patients had pure teratomas. One patient diagnosed with pure teratoma CS2 was positive for miR371, and miR371 expression persisted until RPLND was performed. Histological diagnosis after RPLND showed metastasis of embryonal carcinoma, as mentioned above. The second teratoma patient had pure teratoma CS1, with GCNIS in the specimen. This patient had miR371 above the threshold before orchiectomy, but miR371 declined to values within the normal range after orchiectomy.

We did not perform experiments designed to investigate the in vivo half-life of miR371 and our material includes few serial samples within the first week after orchiectomy, but the initial in vivo half-life of miR371 was estimated to be less than 5 h using non-linear regression analysis of three confirmed CS1 patients with at least two samples post-orchiectomy (Supplementary Figure 4).

### AFP and hCGβ

**Figure 4:**
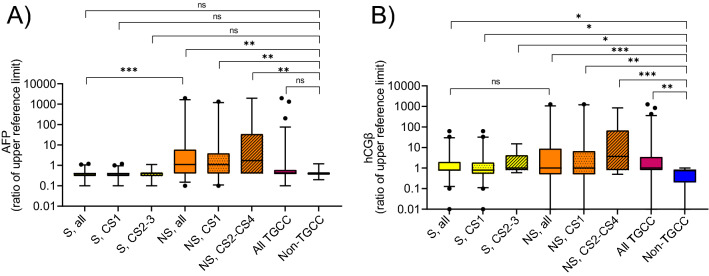
Expression of AFP and hCGβ across the histological sub-types and clinical stages. Shown are mean values with 95% confidence interval. *S* seminomas, *NS* non-seminomas, *TGCC* testicular germ cell cancer, *TGCCC* patients included with suspected TGCC, but diagnosed with other benign and malignant conditions; Healthy males, blood donors. ***P < 0.001; **P < 0.01; *P < 0.05, ^ns^P > 0.05.

The classical markers AFP and hCG_β_ had levels above the thresholds for 21% and 42% of the patients with TGCC, respectively. Seminomas CS1 had the lowest expression with only 33% expressing hCG_β_. Among the non-seminomas CS1, 50% expressed AFP and 46% expressed hCG_β_ above the reference thresholds. The expression of AFP and hCG_β_ before orchiectomy across the histological subtypes and clinical stages are shown in Fig. 4. The expression of LDH can be found in Supplementary Figure 5.

### Stability of miR371 in serum

MiR371 levels in serum separated from the blood cells and incubated at room temp showed marked decrease. The decrease was statistically significant after 5 h for miR371 (*P* = 0.038, Supplementary Figure 6A) and after 3 h for miR30b (*P* = 0.015, Supplementary Figure 6C) at RT. Variation in degradation among the samples were noted. Storage of serum at 4 °C after separation inhibits degradation (Supplementary Figure 6B) and Supplementary Figure 6D). The decrease in miR371 at 4 °C was statistically significant after 4 h (*P* = 0.027), but not for miR30b (*P* = 0.54). After 30 h at 4 °C, the decrease in both miR371 and miR30b were statistically significant (*P* < 0.001), but less than for the samples at RT. The mean miR371 ratios after 4 h at RT and 4 °C were 0.7 (SD = 0.13) and 0.8 (SD = 0.08), respectively. After 30 h at RT and 4 °C, the mean miR371 ratios were 0.2 (SD = 0.05) and 0.7 (SD = 0.15), respectively.

## Discussion

In the current study, we have established analysis of serum miR371 in TGCC using RT-ddPCR and compared this protocol to the RT-qPCR protocol used by others^20–22,24,37–39^. The results show that RT-ddPCR has advantages over RT-qPCR and clearly has potential as a diagnostic test for miR371. Our study with prospectively included patients verify the results from other published reports stating that circulating miR371 is a sensitive and specific biomarker for detection of viable TGCC cells.

RT-qPCR are the most commonly used method to quantify microRNAs when a limited number of microRNAs are to be analysed. The method is well known, the instruments are available at most laboratory facilities, the 384-format is relatively cost-effective and yield high-throughput of samples. In order to increase the sensitivity further, a pre-amplification step was added for RT-qPCR detection of miR371 from serum or plasma^26,27^. RT-ddPCR has emerged as a new and sensitive PCR based method for detection of microRNAs^28,30^. In ddPCR, the sample with target is partitioned into thousands of small oil droplets where the PCR amplification is carried out. The number of target copies are calculated based on the number of positive droplets which contain at least one copy of the target molecule, relative to total number of droplets according to the Poisson distribution^40–42^. We have here compared the performance of ddPCR without pre-amplification and RT-qPCR with pre-amplification. Thresholds were established for RT-qPCR and RT-ddPCR for calling of positive samples based on LOD experiments and the miR371 levels from the healthy blood donors. For RT-qPCR, our threshold is in line with the work of Mego et al.^43^.

According to our findings both methods perform well, but we found RT-ddPCR superior for analysis of microRNAs in serum as the pre-amplification step can be avoided and the output (copies/µL PCR reaction) can easily be converted to copies/µL serum without the use of a standard curve. Analysis of microRNAs in serum or plasma near the LOD is challenging and demands strict monitoring of the protocols. There are two main approaches for detection and measurement of circulating miR371 in serum or pIasma. One is the ΔΔCt-method as used by amongst others Dieckmann et al.^20^ and the second is reporting of raw Cq-values as used by Nappi et al.^24^. The ΔΔCt-method demands normalization to an endogenous miR and a reference sample. The use of this method has been debated^44^. We adhere to the strategy of Nappi et al. where the endogenous control microRNA is not used for normalising purposes, but as a control of sample and protocol quality and performance. High levels of miR30b, the microRNA used as endogenous control, in serum and plasma can be a result of haemolysis during phlebotomy or sample processing, rather than endogenous variation^45,46^. Although Lobo et al. find that their magnetic bead-based RNA extraction protocol^47^ reduces the problem of haemolysis due to haemoglobin’s PCR inhibitory effect, it is undisputable that miR30b and other endogenous microRNAs present in the cells of the human blood, will be released into the plasma compartment if blood cells are damaged during phlebotomy, sample processing or sample storage. As a consequence of this, blood samples should be drawn according to the standard procedures of good hospital practice, i.e. avoid strong haemolysis and phlebotomy downstream of an intravenous infusion site and avoid delayed time to processing and analysis^48^. Regarding PCR inhibition, ddPCR has been shown to have the advantage over traditional PCR that it is less prone to PCR inhibitors due to the dilution and partition into droplets prior to PCR^49^.

TGCC is the most common malignancy among young men with a high cure rate and with a growing number of long-term survivors. The focus is now on how to minimise long-term treatment-related toxicity while maintaining the high cure rate. The majority of patients with localised disease is, in order to minimise the risk of long term and late side effects, followed by surveillance only. However, a considerable portion of stage I patients who are under surveillance will relapse and need three to four cycles of cisplatin-based chemotherapy^50^. For CS 1 patients, new sensitive and specific biomarkers are instrumental tools in guiding clinicians to select patients for adjuvant chemotherapy. Another challenge in clinical practice is how to separate benign from malignant tumours in the case of slightly enlarged nodes detected at CT or MRI scans. MiR371 has been shown to be a very promising circulating biomarker in TGCC^8–14^, but it has yet to be implemented in the diagnostic and clinical use. Regarding the classical markers in TGCC, seminomas do per definition not express AFP and hCG_β_ is expressed at low pathological levels in only ~ 30% of patients^14^. We and others find ~ 90% of seminomas to express miR371. In contrast to Dieckmann et al., we find the sensitivity of miR371 to be equal for detection of seminomas and non-seminomas in CS1^20^, but our cohort is smaller than the one studied by Dieckmann. In addition to being sensitive in detecting TGCC, serum miR371 is a good marker for treatment response. For 97.9% of the CS1 cases, miR371 drops after orchiectomy. miR371 also drops in 85.7% of the metastatic cases, but not to within the normal range. The ratio between pre- and post-orchiectomy miR371 are lower for metastatic cases than for CS1. Of the two CS1 patients with no decline in miR371, one had two borderline enlarged lymph nodes with no change of size and are still considered CS1 and under surveillance. The other shows no signs of metastases and is still considered CS1 30 months after orchiectomy. Seminoma patients classified as CS1 have good prognosis and are preferably followed by surveillance only. In this setting, miR371 can be instrumental in detecting microscopic disease after orchiectomy or relapse. The SWENOTECA guidelines for management of TGCC includes re-staging 6–8 weeks for the CS1 and CS2A marker negative (Mk-) non-seminomas, which may be replaced by miR371 in the future. None of the patients included with suspected TGCC, but diagnosed with other diagnoses after orchiectomy had miR371 levels above the threshold. Our study includes only a limited number of non-germ cell testicular cancer cases, thus the specificity and NPV must be interpreted with caution.

MiR371 with its high sensitivity and short in vivo half-life can enable confirmation of the clinical stage within days after orchiectomy. Our results indicate an initial in vivo half-life shorter than 5 h for miR371, which is in line with findings from others^51^. The in vivo half-life of AFP and hCG_β_ are 5–7 days and 24–36 h, respectively. The classical biomarkers in TGCC thus have considerably longer half-life, and they therefore do not reflect treatment response as early as miR371. The preliminary results presented here indicate that miR371 show a two-phase decay with a slower phase following the first rapid decay. Our experiment was not designed to study the half-life of miR371 and have a limited number of cases with samples at more than one timepoint within the first week after orchiectomy. Repeated blood sampling within the first 24 h to establish the rapid phase of decay followed by multiple samples within the first week after orchiectomy to investigate the possible second, slow phase of decay needs to be performed. Such an experiment will be inconvenient for the patient, as discussed by Radke et al.^51^.

Serum miR371 is suitable as a biomarker as it is detected at higher levels in TGCC patients compared to healthy individuals, samples can be obtained and miR371 detected by standard laboratory methods and it has short half-life in circulation in vivo. However, we show that in a clinical setting, the in vitro degradation of serum miR371 should be taken into consideration as we demonstrate statistically significant degradation after 5 h at room temperature from serum separation. Similar findings for other microRNAs have been shown^27,33–35^. Storage at 4 °C slows the degradation, but is still statistically significant after 4 h. Our stability study includes a limited number of samples and our findings need to be verified.

In conclusion, we demonstrate that RT-ddPCR without pre-amplification performs well for detection of serum miR371. Our study verifies miR371 as a good serum marker with sensitivity superior to the classical markers AFP and hCG_β_ for detection and monitoring of TGCC, except for the teratomas. A large proportion of the TGCC patients are followed by surveillance only after orchiectomy and the use of miR371 will be a valuable addition to imaging in the clinical monitoring of these patients, and may help to select patients for adjuvant chemotherapy. We propose the next step in implementation of miR371 as a biomarker in TGCC to be a prospective study where serum miR371 is used in addition to imaging and established risk factors for identification of the CS1 patients who should be offered adjuvant chemotherapy.

## Methods

### Study participants

Patients were included through the SWENOTECA-MIR study. The inclusion criteria were suspected TGCC at the time of orchiectomy, age 18–70 years and no prior history of cancer. The inclusion period was February 2016 to November 2020, and we included patients at several centres in Norway and Sweden (Supplementary Figure 1). Clinical information regarding age, histological type, tumour diameter, clinical stage according to the modified Royal Marsden classification^52^, localization of metastases, treatment given and expression of classical biomarkers AFP, LDH and hCG_β_ were recorded. In order to establish a preliminary reference level, we included samples from 50 age matched, healthy male blood donors.

### Blood sampling and RNA isolation

**Figure 5:**
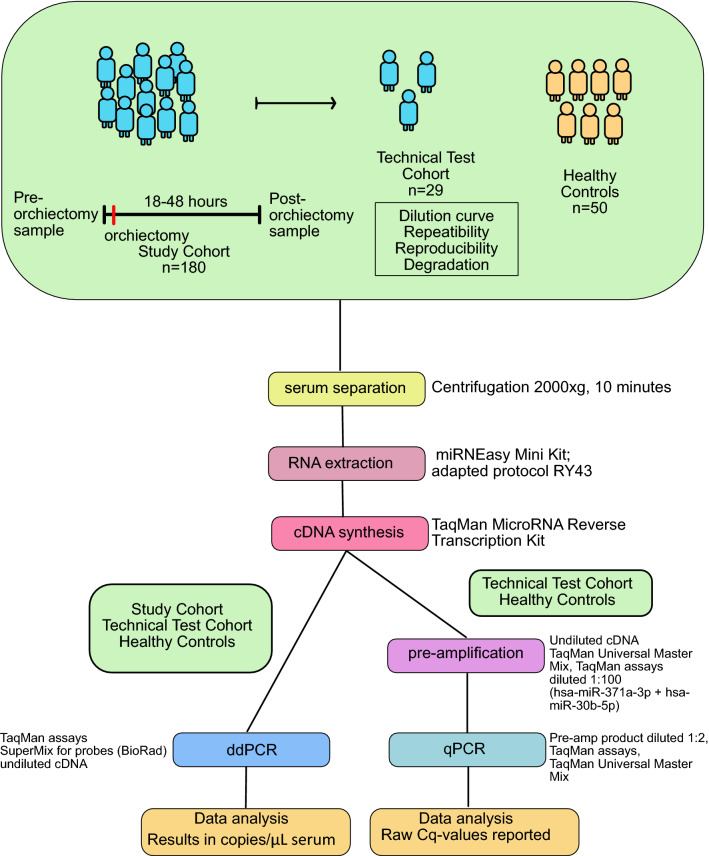
Flow chart summarizing the flow of patients through the study and the laboratory methods used in each step.

Serum samples for study purposes were collected at the same time as standard clinical blood samples. The baseline sample was collected prior to orchiectomy, preferably less than 24 h prior to surgery. The post orchiectomy samples were collected 18–48 h after surgery (Fig. 5). The standard markers AFP, hCG_β_ and LDH were measured at the same timepoints. Blood was collected by standard venepuncture technique into 10 mL Vacutainer tubes with clot activator (BD P/N 367896). The samples were allowed to clot for 1 h prior to centrifugation at 2000×*g* for 10 min. Serum was carefully removed, aliquoted and frozen immediately at – 80 °C.

Prior to RNA extraction, serum was thawed, and haemolysis was assessed spectrophotometrically at 414 nm for all serum samples ^45^. Total RNA including small RNAs was extracted from 200 µL serum using the miRNEasy (Qiagen P/N 217004) and the supplementary protocol RY43 (Version Feb-11). Briefly, five volumes of Qiazol Lysis Reagent were added and vortexed for homogenization. Prior to addition of 1 volume of chloroform and centrifugation, we added 3 µg glycogen (Fisher Scientific, P/N R0551) as carrier. The upper phase was carefully transferred to a new tube where 1.5 volumes of 96% ethanol was added and column purification performed. RNA was eluted in 30 µL nuclease-free water.

### Reverse transcription (RT)

For the hydrolysis probe-based detection of miR-371a-3p, cDNA was synthesised using a pool containing equal amounts of the specific RT-primers from TaqMan assays miR-371a-3p (miR371, P/N 4427975, assay ID 002124, ThermoFisher Scientific) and miR-30b-5p (miR30b, P/N 442795 assay ID 000602). Each reaction consisted of 5 µL RNA template, 0.15 µL 100 mM dNTP-mix, 1.0 µL Multiscribe 50 U/µL, 1.5 µL 10 × RT-buffer, 0.19 RNase Inhibitor (20 U/µL), 3 µL primer pool and 4.16 µL nuclease-free water. All components were from the TaqMan™MicroRNA Reverse Transcription Kit (P/N 4366596, Thermo Fisher Scientific). Reaction conditions were 16 °C for 30 min, 42 °C for 30 min, 85 °C for 5 min before cooling to 4 °C.

### QPCR with pre-amplification

The preamplification step was performed with a pool of the 20 × TaqMan microRNA Assays diluted to 0.2 × in TE-buffer (10 mM Tris, 0.1 mM EDTA, pH = 8.0). The pre-amp reaction was prepared by combining 8.0 µL 2 × TaqMan Universal Mastermix, no UNG (P/N 4440040), 4.0 µL 0.2 × Assay Pool and 4.0 µL undiluted cDNA. The cycling conditions were 95 °C for 10 min, followed by 14 cycles of 95 °C for 15 s, 60 °C for 4 min, and hold at 4.0 °C. Further, qPCR was performed in single plex by combining 0.5 µL TaqMan microRNA Assay (20 ×), 1.0 µL pre-amp product diluted 1:2, 5.0 µL TaqMan Universal Mastermix (2 ×) and 3.5 µL nuclease-free water in 384-well plates. The cycling conditions on the LightCycler 480 Real-Time PCR System were 95 °C for 10 min, followed by 45 cycles of 95 °C for 15 s and 60 °C for 4 min. Technically, we called a sample as positive when Cq < 35 and the amplification curve was exponential with the expected fluorescence intensity.

### DdPCR

cDNA was synthesised as described for RT-qPCR. No pre-amp was performed. Undiluted cDNA, 3 µL, was mixed with 10.0 µL Supermix for Probes (P/N 186-024, Bio-Rad), 1.0 µL TaqMan microRNA Assays (20 ×) and 6.0 µL nuclease-free water. The 96-well plate was sealed and droplets generated by the Automated Droplet Generator and Droplet Generation Oil for Probes (P/N 1864110, Bio-Rad). The cycling condition was 15 min at 95 °C, followed by 40 cycles of 95 °C for 15 s and 60 °C for 1 min, then 98 °C for 10 min and cooling to 4 °C. Ramp rate was 2 °C/s. A gradient of annealing temperature 56–62 °C was tested. The separation of positive and negative droplets was good at 60 °C, which is the recommended annealing temperature for both Supermix for Probes and the Small RNA Assays (Supplementary Information and Data 1). Droplets were analysed using the QX200 Droplet Reader and the QuantaSoft Analysis Pro software (Bio-Rad). The thresholds for positive droplets were amplitude 3500 for miR371 and 4000 for miR30b across all samples. Triplicate wells were analysed for all samples.

The miR371 and miR30b concentrations from RT-ddPCR are presented as copies/µL serum if not otherwise stated and were calculated from the instrument output given in copies/µL PCR reaction (see Supplementary Information and Data 2).

### Linearity, limit of blank (LOB), limit of detection (LOD) and limit of quantitation (LOQ)

We performed two parallel dilution experiments to assess the linearity of the miR371 assay in RT-qPCR with pre-amplification and ddPCR without pre-amplification. First, we performed a two-fold serial dilution of RNA from a patient sample with known positive miR371 expression in nuclease-free water as described by Hindson et al.^28^. Three parallel dilution series were made, cDNA was synthesised and thereafter analysed by qPCR with pre-amplification and ddPCR (without pre-amplification). Second, for further assessment of the performance and precision at low concentrations we performed an additional dilution series at the lower range. cDNA was diluted two-fold and ten qPCR and ddPCR reactions were carried out for each dilution. In order to assess the reproducibility of the miR371 assay towards the LOD, we selected 13 samples with negative or low miR371 levels and extracted RNA on a different day and measured miR371 a second time. Measurements by ddPCR and qPCR were performed from the same cDNA, and the results from the two runs (day 1 vs. day 2) were compared. The repeatability of the protocol from RNA extraction through qPCR with pre-amplification and ddPCR was tested by repeated measurement of miR371 in samples from three patients with different levels of miR371 expression.

LOB and LOD for RT-ddPCR were established according to Armbruster et al.^53^.

$$\begin{aligned} {\text{LOB}}_{{{99}}} &= {\text{Mean}}\,{\text{miR371}}_{{{\text{Blood}}\,{\text{donors}}}} + { 1}.{96}\,{\text{SD}},\,{\text{at}}\,{\text{the}}\,{99}\% \,{\text{confidence}}\,{\text{level}}. \hfill \\ {\text{LOD}}_{{{99}}} &= {\text{LOB }} + {3 } \times {\text{ SD}}_{{{\text{Blood}}\,{\text{donors}}}} \hfill \\ \end{aligned}$$

LOQ was defined as the lowest concentration that could be detected with CV ≤ 25%^54^.

### In vitro stability of miR371

The stability of miR371 in serum were initially tested at room temperature (RT) by processing serum according to our standard protocol, prior to incubation at room temperature. Aliquots were extracted at baseline and after 1 h, 3 h, 5 h, 24 h, 30 h and 48 h. Aliquots of 200 µL serum were added directly to Qiazol lysis reagent and stored frozen at – 80 °C until extraction. The stability of miR371 in serum stored at 4 °C after separation were performed with aliquots sampled at baseline, after 4 h and 30 h.

### Ethics statement and statistics

The patient samples analysed have been included through the SWENOETCA-MIR study which has been approved by the Regional Ethics Committee (REC Central Norway 2015/1475) and Regional Ethics Committee (REC Stockholm 2018/1730-31). Written, informed consent was obtained from all patients at inclusion, in accordance with the Declaration of Helsinki.

Differences among categorical variables were calculated using the χ^2^ test. A two-sided Mann–Whitney U test was used to assess differences between mean of groups. All tests were two-sided and significance was assumed at *P* < 0.05. The association between log transformed miR371 and tumour size was tested using linear regression, presented with R^2^. Medians were compared using the non-parametric test for two independent samples. The statistical analyses were performed using SPSS version 25 (IBM Corporation) and graphs were produced using GraphPad Prism 8 (GraphPad Software, LLC). The figures were prepared using Affinity Designer (version 1.9.0.932, Serif). Sensitivity, specificity, positive prediction value (PPV) and negative prediction value (NPV) were calculated.

Due to differences in analytical protocols and reference levels at the study centres, AFP and hCG_β_ levels for each patient were calculated as ratios to the upper reference level at the given centre.

## Supplementary Information

Supplementary Informations.

## Data Availability

The data supporting the findings of this study are available from the corresponding author upon reasonable request.

## Abbreviations

hCGβ: Human chorionic gonadotropin type beta
NTC: No-template control
AFP: Alpha-fetoprotein
Cq: Cycle of quantification
CS: Clinical stage
miR371: MicroRNA-371a-3p
LDH: Lactate dehydrogenase
LOB: Limit-of-blank
LOD: Limit-of-detection
LOQ: Limit-of-quantification
PPV: Positive prediction value
RT-ddPCR: Reverse transcriptase digital-droplet PCR
RT-qPCR: Reverse-transcriptase quantitative PCR (also known as real-time-PCR)
RPLND: Retroperitoneal lymph node dissection
TGCC: Testicular germ cell cancer

## References

[1] Williamson SR (2017). The World Health Organization 2016 classification of testicular germ cell tumours: A review and update from the International Society of Urological Pathology Testis Consultation Panel. Histopathology.

[2] Honecker F (2018). ESMO Consensus Conference on testicular germ cell cancer: Diagnosis, treatment and follow-up. Ann. Oncol..

[3] *SWENOTECA X—A Cancer Care Program for Germ Cell Tumours*. www.swenoteca.org. (2020). Accessed 10 Mar 2021.

[4] Hellesnes R (2020). Continuing increased risk of second cancer in long-term testicular cancer survivors after treatment in the cisplatin era. Int. J. Cancer.

[5] Brydoy M (2010). Paternity and testicular function among testicular cancer survivors treated with two to four cycles of cisplatin-based chemotherapy. Eur. Urol..

[6] Brydoy M (2009). Observational study of prevalence of long-term Raynaud-like phenomena and neurological side effects in testicular cancer survivors. J. Natl. Cancer Inst..

[7] Haugnes HS (2009). Pulmonary function in long-term survivors of testicular cancer. J. Clin. Oncol..

[8] Haugnes HS (2018). Hearing loss before and after cisplatin-based chemotherapy in testicular cancer survivors: A longitudinal study. Acta Oncol..

[9] Kvammen O (2016). Long-term relative survival after diagnosis of testicular germ cell tumor. Cancer Epidemiol. Biomark. Prev..

[10] Haugnes HS (2012). Long-term and late effects of germ cell testicular cancer treatment and implications for follow-up. J. Clin. Oncol..

[11] Groot HJ (2018). Risk of solid cancer after treatment of testicular germ cell cancer in the platinum era. J. Clin. Oncol..

[12] Bokemeyer C, Berger CC, Kuczyk MA, Schmoll HJ (1996). Evaluation of long-term toxicity after chemotherapy for testicular cancer. J. Clin. Oncol..

[13] Travis LB (2000). Treatment-associated leukemia following testicular cancer. J. Natl. Cancer Inst..

[14] Dieckmann KP (2019). Serum tumour markers in testicular germ cell tumours: Frequencies of elevated levels and extents of marker elevation are significantly associated with clinical parameters and with response to treatment. Biomed. Res. Int..

[15] Bartel DP (2018). Metazoan microRNAs. Cell.

[16] Suh MR (2004). Human embryonic stem cells express a unique set of microRNAs. Dev. Biol..

[17] Voorhoeve PM (2006). A genetic screen implicates miRNA-372 and miRNA-373 as oncogenes in testicular germ cell tumors. Cell.

[18] Gillis AJ (2013). Targeted serum miRNA (TSmiR) test for diagnosis and follow-up of (testicular) germ cell cancer patients: A proof of principle. Mol. Oncol..

[19] Belge G, Dieckmann KP, Spiekermann M, Balks T, Bullerdiek J (2012). Serum levels of microRNAs miR-371-3: A novel class of serum biomarkers for testicular germ cell tumors?. Eur. Urol..

[20] Dieckmann KP (2019). Serum levels of microRNA-371a-3p (M371 test) as a new biomarker of testicular germ cell tumors: Results of a prospective multicentric study. J. Clin. Oncol..

[21] Syring I (2015). Circulating serum miRNA (miR-367-3p, miR-371a-3p, miR-372-3p and miR-373-3p) as biomarkers in patients with testicular germ cell cancer. J. Urol..

[22] Murray MJ (2016). A pipeline to quantify serum and cerebrospinal fluid microRNAs for diagnosis and detection of relapse in paediatric malignant germ-cell tumours. Br. J. Cancer.

[23] Belge G (2021). Serum levels of microRNA-371a-3p are not elevated in testicular tumours of non-germ cell origin. J. Cancer Res. Clin..

[24] Nappi L (2019). Developing a highly specific biomarker for germ cell malignancies: Plasma mir371 expression across the germ cell malignancy spectrum. J. Clin. Oncol..

[25] Leao R (2018). Serum miRNA predicts viable disease after chemotherapy in patients with testicular nonseminoma germ cell tumor. J. Urol..

[26] Murray MJ (2011). Identification of microRNAs from the miR-371~373 and miR-302 clusters as potential serum biomarkers of malignant germ cell tumors. Am. J. Clin. Pathol..

[27] Mitchell PS (2008). Circulating microRNAs as stable blood-based markers for cancer detection. Proc. Natl. Acad. Sci. USA.

[28] Hindson CM (2013). Absolute quantification by droplet digital PCR versus analog real-time PCR. Nat. Methods.

[29] Robinson S (2018). Droplet digital PCR as a novel detection method for quantifying microRNAs in acute myocardial infarction. Int. J. Cardiol..

[30] Campomenosi P (2016). A comparison between quantitative PCR and droplet digital PCR technologies for circulating microRNA quantification in human lung cancer. BMC Biotechnol..

[31] Strain MC (2013). Highly precise measurement of HIV DNA by droplet digital PCR. PLoS ONE.

[32] Murray MJ (2018). “Future-proofing” blood processing for measurement of circulating miRNAs in samples from biobanks and prospective clinical trials. Cancer Epidemiol. Biomark. Prev..

[33] Glinge C (2017). Stability of circulating blood-based microRNAs—pre-analytic methodological considerations. PLoS ONE.

[34] Koberle V (2013). Differential stability of cell-free circulating microRNAs: Implications for their utilization as biomarkers. PLoS ONE.

[35] Yamada A (2014). Technical factors involved in the measurement of circulating microRNA biomarkers for the detection of colorectal neoplasia. PLoS ONE.

[36] Bustin S (2015). Variability of the reverse transcription step: Practical implications. Clin. Chem..

[37] Lobo J, Gillis AJM, Jeronimo C, Henrique R, Looijenga LHJ (2019). Human germ cell tumors are developmental cancers: Impact of epigenetics on pathobiology and clinic. Int. J. Mol. Sci..

[38] Dieckmann KP (2012). MicroRNAs miR-371-3 in serum as diagnostic tools in the management of testicular germ cell tumours. Br. J. Cancer.

[39] van Agthoven T, Looijenga LHJ (2017). Accurate primary germ cell cancer diagnosis using serum based microRNA detection (ampTSmiR test). Oncotarget.

[40] Dube S, Qin J, Ramakrishnan R (2008). Mathematical analysis of copy number variation in a DNA sample using digital PCR on a nanofluidic device. PLoS ONE.

[41] Saiki RK (1988). Primer-directed enzymatic amplification of DNA with a thermostable DNA polymerase. Science.

[42] Vogelstein B, Kinzler KW (1999). Digital PCR. Proc. Natl. Acad. Sci. USA.

[43] Mego M (2019). Clinical utility of plasma miR-371a-3p in germ cell tumors. J. Cell. Mol. Med..

[44] Spiekermann M, Dieckmann KP, Balks T, Bullerdiek J, Belge G (2015). Is relative quantification dispensable for the measurement of microRNAs as serum biomarkers in germ cell tumors?. Anticancer Res..

[45] Myklebust MP (2019). Quantitative PCR measurement of miR-371a-3p and miR-372-p is influenced by hemolysis. Front. Genet..

[46] Shah JS, Soon PS, Marsh DJ (2016). Comparison of methodologies to detect low levels of hemolysis in serum for accurate assessment of serum microRNAs. PLoS ONE.

[47] Lobo J (2019). Identification and validation model for informative liquid biopsy-based microRNA biomarkers: Insights from germ cell tumor in vitro vivo and patient-derived data. Cells.

[48] Boeckel JN (2013). Heparin selectively affects the quantification of microRNAs in human blood samples. Clin. Chem..

[49] Dingle TC, Sedlak RH, Cook L, Jerome KR (2013). Tolerance of droplet-digital PCR vs real-time quantitative PCR to inhibitory substances. Clin. Chem..

[50] Daugaard G (2014). Surveillance for stage I nonseminoma testicular cancer: Outcomes and long-term follow-up in a population-based cohort. J. Clin. Oncol..

[51] Radtke A (2018). The novel biomarker of germ cell tumours, micro-RNA-371a-3p, has a very rapid decay in patients with clinical stage 1. Urol. Int..

[52] Peckham MJ, McElwain TJ, Barrett A, Hendry WF (1979). Combined management of malignant teratoma of the testis. Lancet.

[53] Armbruster DA, Pry T (2008). Limit of blank, limit of detection and limit of quantitation. Clin. Biochem. Rev..

[54] Milosevic D (2018). Applying standard clinical chemistry assay validation to droplet digital PCR quantitative liquid biopsy testing. Clin. Chem..

