# Supplementary material for: Serum miR371 in testicular germ cell cancer before and after orchiectomy, assessed by digital-droplet PCR in a prospective study
Source: Sci Rep. 2021 Aug 2;11:15582. doi: 10.1038/s41598-021-94812-2 (PMC8329070; doi:10.1038/s41598-021-94812-2)
Supplement: Supplementary file 1 — Supplementary Informations. [file 41598_2021_94812_MOESM1_ESM.pdf]

Supplementary Information and Data

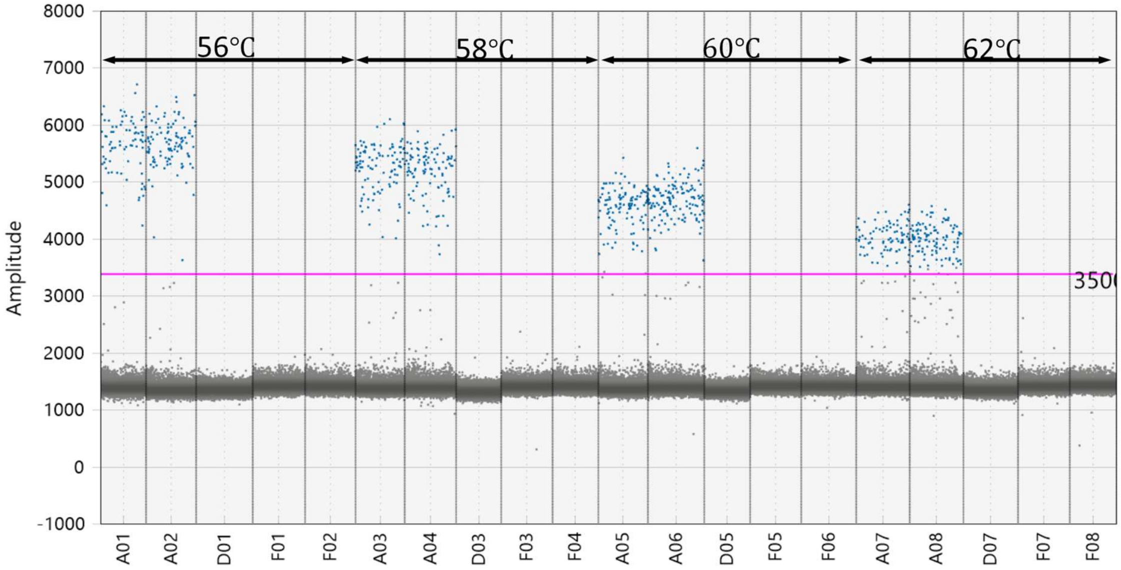

Supplementary Information and Data 1: Annealing temperature gradient for miR371 ddPCR. Wells A1-8 contain a known positive sample, wells D1-7 contain NTC and wells F1-7 contain a known negative sample. The samples were analysed by ddPCR with the indicated annealing temperature.

Supplementary Information and Data 2: Formula for calculation of copies/µL serum from the ddPCR output in copies/ µL PCR reaction

$$SC = C_{PCR} * V_{PCR} * \frac{cDNA\ RXvol}{cDNA\ input\ vol} * DF * \frac{EV}{RNA} / S_{vol} \quad [1]$$

simplified in our protocol:

$SC_{miR371} = C_{PCR} * 3$

$SC_{miR30b} = C_{PCR} * 150$

SC= Serum concentration (copies/ µL serum)

C_{PCR}= Results from PCR, Copies/ µL PCR

V_{PCR}= Total volume PCR (20 µL)

cDNA RX vol= reaction volume cDNA synthesis (15 µL)

cDNA input vol= input volume of cDNA into ddPCR (3 µL)

DF = Dilution factor of cDNA (1 for miR371, 50 for miR30b)

EV= Elution volume from RNA extraction (30 µL)

RNA= RNA volume into cDNA synthesis (5 µL)

S_{vol}= Serum volume taken into RNA extraction (200 µL)

Reference

1. van Ginkel, J.H., Huibers, M.M.H., van Es, R.J.J., de Bree, R., and Willems, S.M., *Droplet digital PCR for detection and quantification of circulating tumor DNA in plasma of head and neck cancer patients.* BMC Cancer, 2017. **17**(1): p. 428.

Supplementary Information and Data 3: Raw Cq-values for repeated RT-qPCR analysis of a sample with miR371 close to the presumed LOD.

Sample	Replicate I	Replicate II	Replicate III	Replicate IV	Replicate V
Sample 1	30.75	29.93	30.03	29.15	30.49
Sample 2	30.80	>40	31.24	30.31	30.98
Sample 3	30.76	31.06	29.98	>40	29.89

Supplementary Information and Data 4: RT-ddPCR for miR371: LOQ estimation

Sample	Measured copies/µL serum	SD	CV(%)
**D1**	5.553	0.631	11.3
**D2**	2.859	0.569	19.0
**D3**	1.570	0.266	**16.9**
**D4**	0.850	0.253	29.8

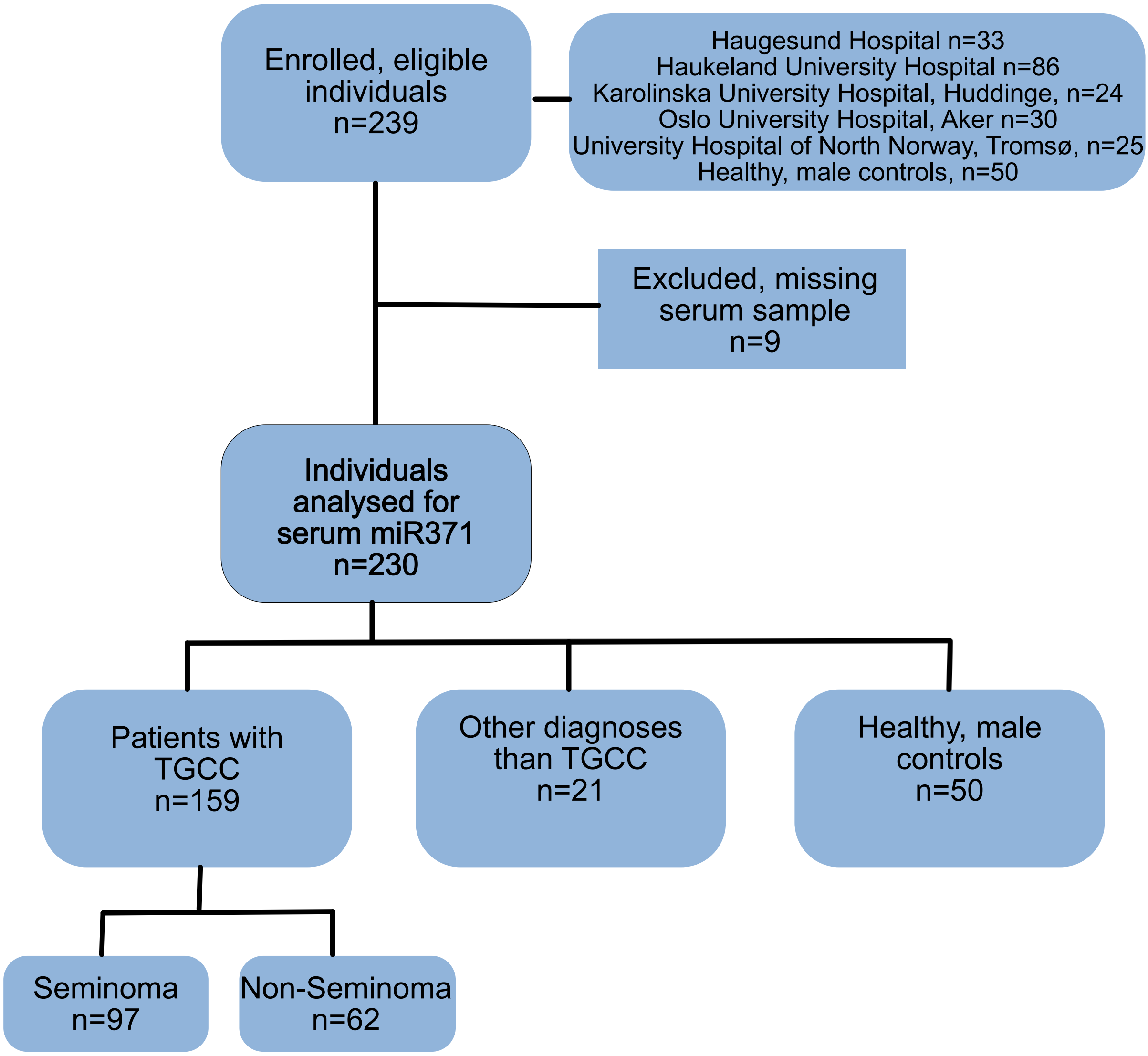

Supplementary Figure 1: Chart showing the enrolment and sub-types of patients through the study.

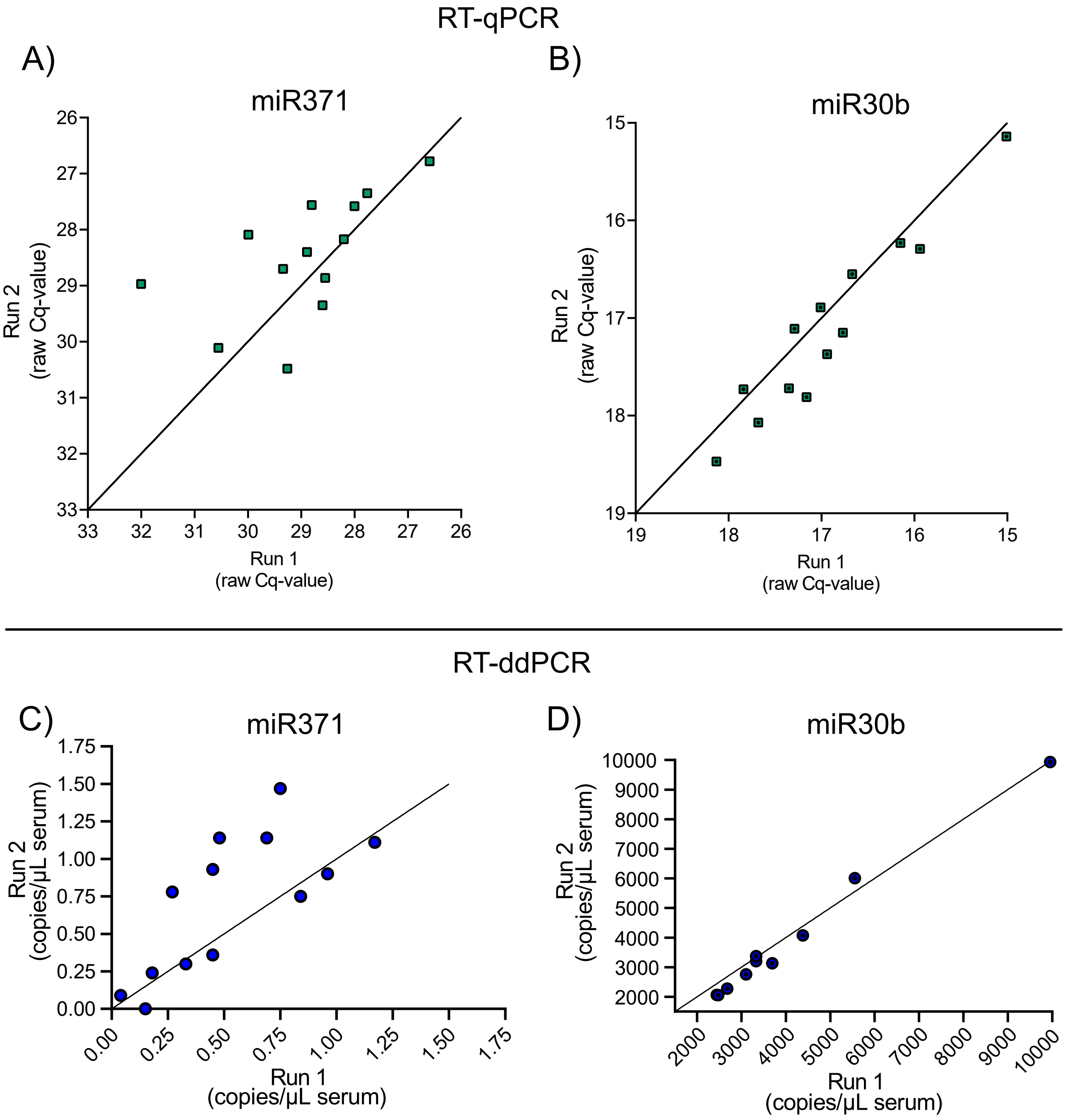

Supplementary Figure 2**:** Reproducibility of miR371 and miR30b quantification by RT-qPCR (A and B) and RT-ddPCR (C and D). RNA extraction, cDNA synthesis and target quantification were performed on two different days. The results were plotted against each other. Shown is also the line of identity. Note that the direction of the axes is reversed for RT-qPCR.

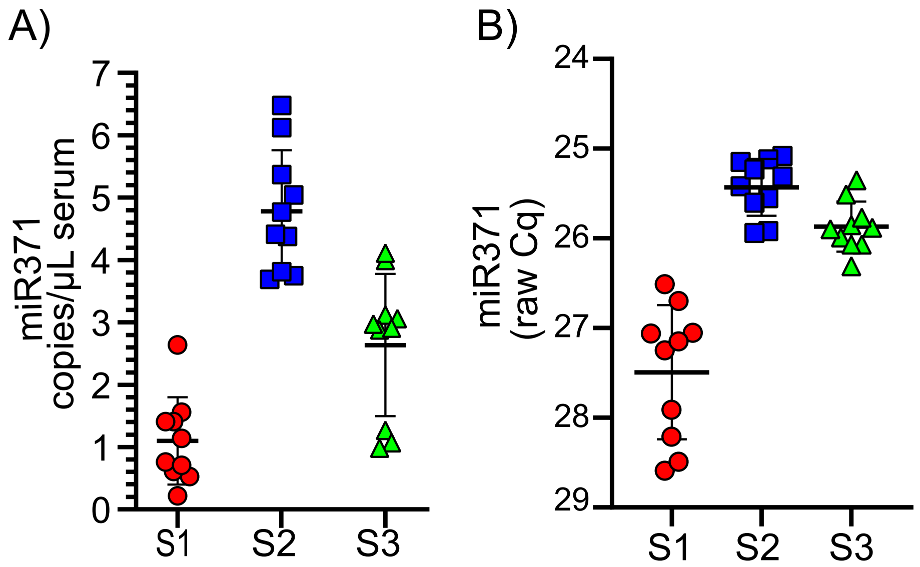

Supplementary Figure 3: The repeatability of miR371 quantification at different concentrations. RNA from three different patient samples (S1, S2 and S3) were extracted ten times at the same day and miR371 quantified by A) RT-ddPCR and B) RT-qPCR. The results are shown as mean value with SD. Note that the direction of the axes is reversed for RT-qPCR.

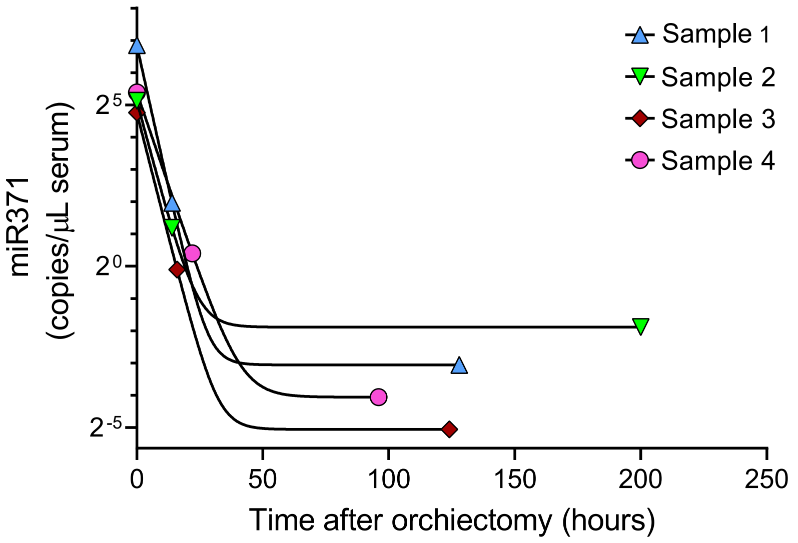

	Sample 1	Sample 2	Sample 3	Sample 4
Estimated half-life	2.8	3.4	3.3	4.4

Supplementary Figure 4: Estimation of *in vivo* miR371 degradation.
Shown are the measured miR371 levels for each patient plotted against time in hours after orchiectomy. The half-life of miR371 was estimated from four confirmed CS1 by non-linear regression curve fit. The half-life estimates are given in hours.

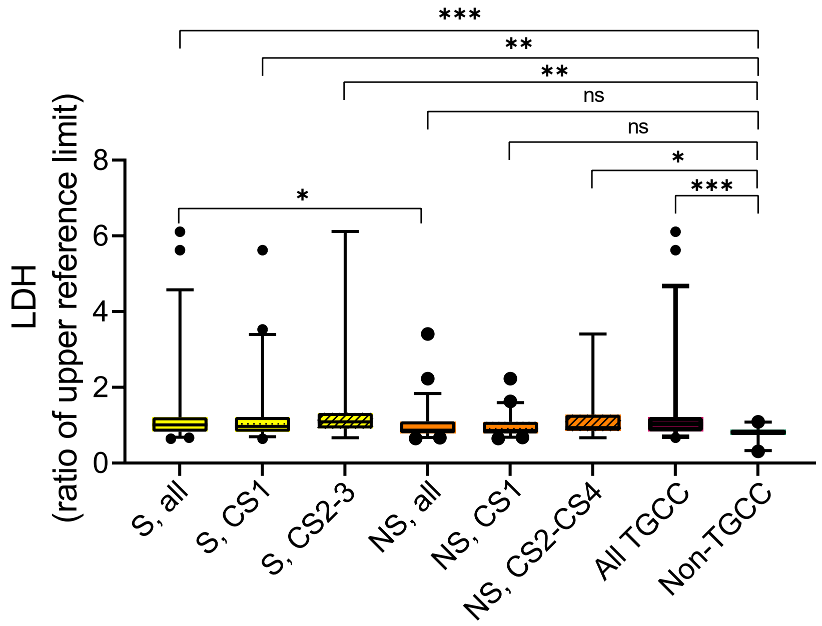

Supplementary Figure 5: Lactate dehydrogenase expression across the histological sub-types and clinical stages.

S, seminomas; NS,Non-seminomas; TGCC, Testicular Germ Cell Cancer; TGCCC, patients included with suspected TGCC, but diagnosed with other benign and malignant conditions;

Healthy males, blood donors. ***: P<0.001, **: P<0.01, *: P<0.05, ns: P>0.05.

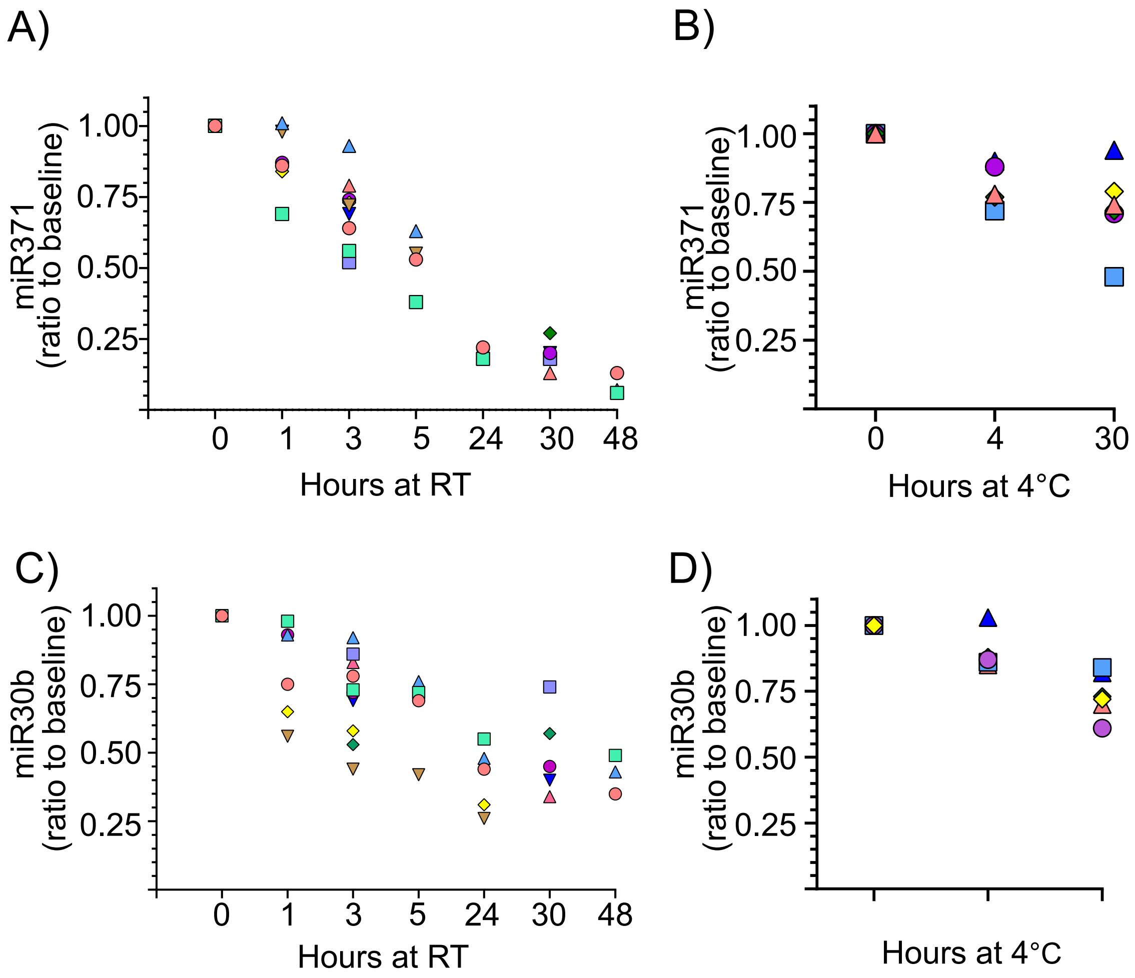

***Supplementary Figure 6*: Degradation of serum miR371 and miR30b *in vitro*. Serum aliquots were stored at RT (A) and 4℃ (B) and frozen at defined timepoints. RNA was extracted and miR371 measured. miR371 (copies/µL serum) at the given timepoint was calculated as the ratio to miR371 at baseline (0 hours). Each time series was from a different patient and is indicated by different colours and shapes. RT, room temperature.
